# Significant enhancement of magnetoresistance with the reduction of particle size in nanometer scale

**DOI:** 10.1038/srep20351

**Published:** 2016-02-03

**Authors:** Kalipada Das, P. Dasgupta, A. Poddar, I. Das

**Affiliations:** 1 CMP Division, Saha Institute of Nuclear Physics, 1/AF, Bidhannagar, Kolkata 700 064, India

## Abstract

The Physics of materials with large magnetoresistance (MR), defined as the percentage change of electrical resistance with the application of external magnetic field, has been an active field of research for quite some times. In addition to the fundamental interest, large MR has widespread application that includes the field of magnetic field sensor technology. New materials with large MR is interesting. However it is more appealing to vast scientific community if a method describe to achieve many fold enhancement of MR of already known materials. Our study on several manganite samples [La_1−*x*_Ca_*x*_MnO_3_ (x = 0.52, 0.54, 0.55)] illustrates the method of significant enhancement of MR with the reduction of the particle size in nanometer scale. Our experimentally observed results are explained by considering model consisted of a charge ordered antiferromagnetic core and a shell having short range ferromagnetic correlation between the uncompensated surface spins in nanoscale regime. The ferromagnetic fractions obtained theoretically in the nanoparticles has been shown to be in the good agreement with the experimental results. The method of several orders of magnitude improvement of the magnetoresistive property will have enormous potential for magnetic field sensor technology.

In present days science based society largely depends on several gadgets where magnetic field sensors play crucial role. The primary requirement for magnetoresistive sensor is the large magnetoresistance. Last two decades perovskite manganites was in the fore-front of the experimental research and resulted several thousand research articles with the primary focus on large magnetoresistance (MR). However, in manganites most of the cases large magnetoresistance occurs at very high (several tesla or more) magnetic field. The requirement of the large external magnetic field severely restrict the use of manganites as magnetic field sensor. Our findings in this manuscript show how the MR of the same material can be increased drastically even at the lower magnetic field.

The doped perovskite manganite is generally represented by the formula R_1−*x*_B_*x*_MnO_3_, where ‘R’ is a trivalent rare earth and ‘B’ is a bivalent element. Numerous fascinating properties of the doped manganites were observed depending on the bivalent element B and its doping concentration *x*. In addition to the metal-insulator transition and colossal magnetoresistance, doped manganite compounds usually show a generic phenomenon of charge ordering close to the doping concentrations *x* ~ 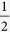, 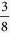 and some other concentrations. The charge-ordering (CO) is the real space ordering of the Mn^3+^ and Mn^4+^ ions and it is generally followed by an antiferromagnetic transition with the lowering of temperature. The high resistive insulating behavior with lowering the temperature below the charge-ordering transition temperature is also a generic behavior of the charge-ordered manganites. In the presence of external perturbations like magnetic field, electric field, x-ray irradiation *etc*., the high resistive insulating state transforms into a low resistive metallic state as a results of destabilization of the charge ordering. The magnetic field-induced destabilization of the charge ordering leads to the gigantic change of the resistance which is known as colossal magnetoresistance. The required magnetic field to destabilize the charge ordered state depends upon the electronic band width of the compounds and the charge-ordered state is more robust for lower band width system. During the last two decades, magnetoresistive properties of several doped perovskite manganites were extensively studied^1^,^2^,^3^,^4^,^5^,^6^,^7^,^8^. To consider manganites for technological purposes such as magnetic field sensor, the high field requirement to melt the charge ordered state is an obstacle. To overcome this, several attempts have been made to reduce the magnetic field requirement. A significant effort to achieve the low field magnetoresistance has been reported by substitute different dopants as well as to make thin films, multilayer flims, core-shell, hetrostructures *etc*.^9^,^10^,^11^,^12^. In contrast to that the detailed systematic study of manganite nanomaterials with the motivation to achieve significant enhancement of MR is lacking in the literature.

In the present article an outstanding route for achieving the significant enhancement of magnetoresistance (MR) that is the particle size dependent enhancement of MR has been discussed in case of the charge-ordered manganite. As a demonstrative example, we have prepared and studied in detail bulk to nanosized La_0.48_Ca_0.52_MnO_3_, La_0.46_Ca_0.54_MnO_3_ and La_0.45_Ca_0.55_MnO_3_ compounds. The melting of the fragile charge ordering in nanoparticles in the presence of comparatively lower external magnetic field plays the vital role in this present study. The phenomena, that is the particle size dependent enhancement of MR also appears to be playing role in several other manganite compounds. The indication of reduction of the required external magnetic field value (H_*C*_) in nano particles of Pr_0.5_Ca_0.5_MnO_3_ (particle size ~40 nm and H_*C*_ = 55 kOe) compared to their respective bulk counterpart (H_*C*_ = 270 kOe) was reported in earlier studies^13^,^14^.

Among the manganite families La_1−*x*_Ca_*x*_MnO_3_ (LCMO) is very well known compound. According to its phase diagram, this compound shows rich physical properties depending on the ‘Ca’ doping concentration ‘*x*’^15^. For lower doping concentration (0.2 < *x* < 0.5), the ferromagnetic ground state is observed. Whereas in the doping region 0.5 < *x* < 0.87 the ground state of the LCMO shows the charge-ordered antiferromagnetic nature with lowering the temperature. The modification of the physical properties, specifically the enhancement of the magnetoresistance and the ferromagnetic fractions in nanoparticles in compared to their bulk counterpart is presented in this report. Good agreement has been obtained between our experimental results and the theoretically simulated data.

## Results and Discussion

The x-ray diffraction measurements of sol-gel prepared samples (bulk and nanoparticles of all series) indicate the successful formation of the single phase nature of all samples (list of all the compounds is mentioned in Table 1). The average size of the nanoparticles of all the compounds were estimated from the Transmission Electron Microscopy (TEM) and Scanning Electron Microscopy (SEM) study. The crystalline particle size from the x-ray line broadening is also calculated using Scherrer’s formula (details are given in the Section-1 of supplementary information part) for lower particle size samples (particle size <100 nm). The particle sizes, determined from SEM, TEM and x-ray line width broadening gives the similar results. The average particle sizes, the annealing temperature and annealing times for the series La_0.48_Ca_0.52_MnO_3_ (LCMO-1) and La_0.46_Ca_0.54_MnO_3_ (LCMO-2) are listed in Tables 2 and 3 respectively.

**Figure 1:**
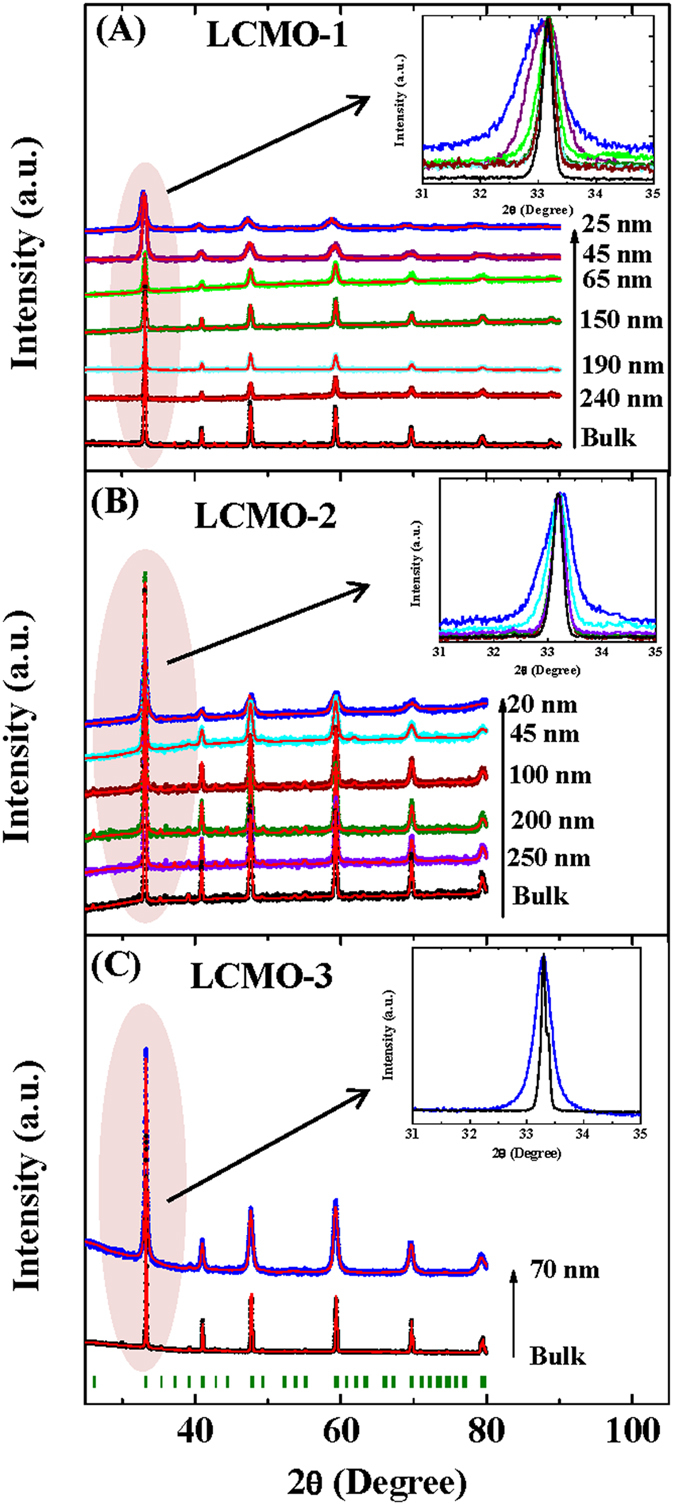
Room temperature x-ray diffraction pattern of the (**A**) LCMO-1, (**B**) LCMO-2 and (**C**) LCMO-3 compounds. Red lines correspond to the profile fitted data. Inset shows the broadening of the x-ray line width due to the reduction of the particle sizes.

In case of the La_0.45_Ca_0.55_MnO_3_ (LCMO-3), one part of the sol-gel prepared powder was annealed at 900 °C for 4 hr. for the preparation of the nanoparticle. The other part was annealed at 1300 °C for 36 hr. for the preparation of the bulk sample. The estimated average particle size of the nanoparticle sample was found to be 70 nm. The profile fitting of the room temperature x-ray diffraction pattern for all compounds were performed considering the ‘*pnma*’ space group. The x-ray diffraction patterns along with the fitted data were shown in Fig. 1.

**Figure 2:**
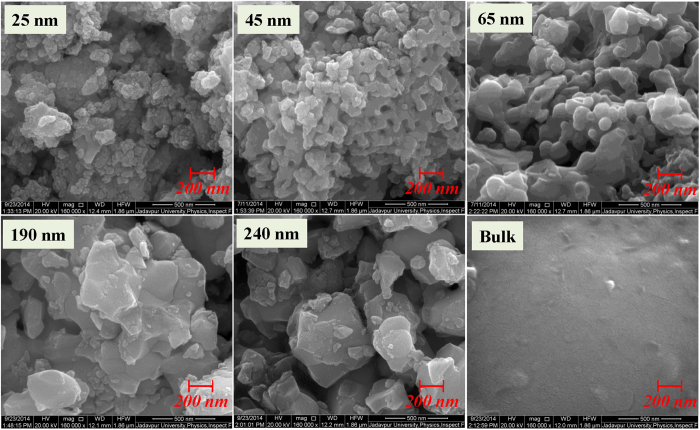
The representative SEM images of nanocrystalline compounds of LCMO-1 series in identical magnification scale including the bulk counterpart.

**Figure 3:**
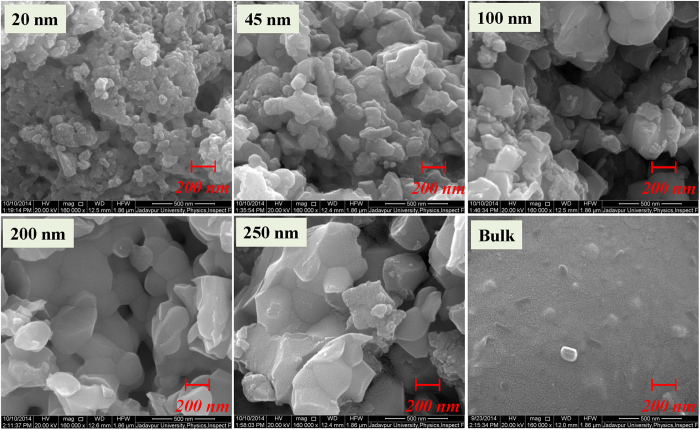
The representative SEM images of nanoparticles of LCMO-2 series in identical magnification scale including the bulk counterpart.

A field emission scanning electron microscope (FEI company, INSPECT F50) and a transmission electron microscope ((FEI company, TECNAI G^2^ F30, S-TWIN) were used to study the surface morphology and the grain size of the synthesized nanoparticles. As a representative picture, the SEM micrographs of nanocrystalline compounds of LCMO-1 and LCMO-2 series are shown in Figs 2 and 3 respectively.

**Figure 4:**
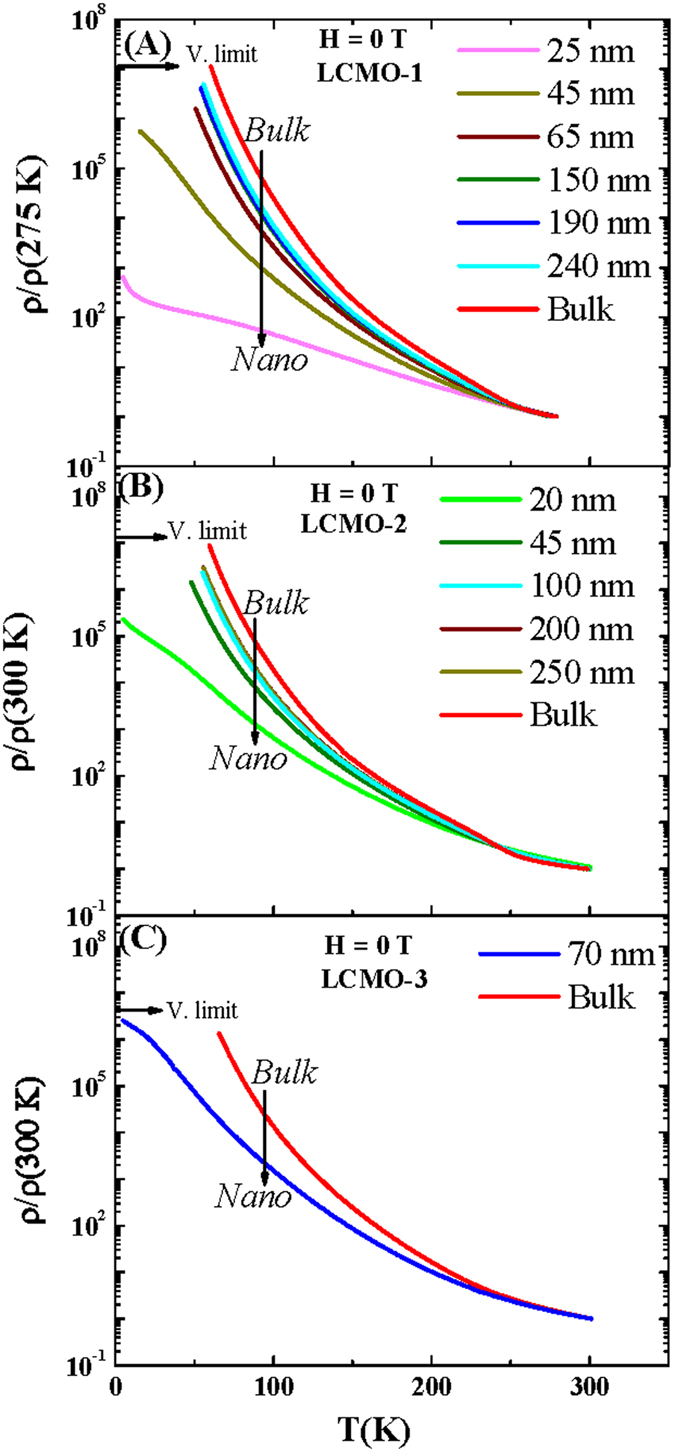
(**A**–**C**) represent the reduced resistivity as a function of temperature for LCMO-1, LCMO-2 and LCMO-3 compound respectively having different particle sizes in the absence of external magnetic field. Figures highlight that the resistivity is decreases with reduction of particle sizes (reduction of the resistivity from bulk to nanoparticles is indicated by the downward arrors).

**Figure 5:**
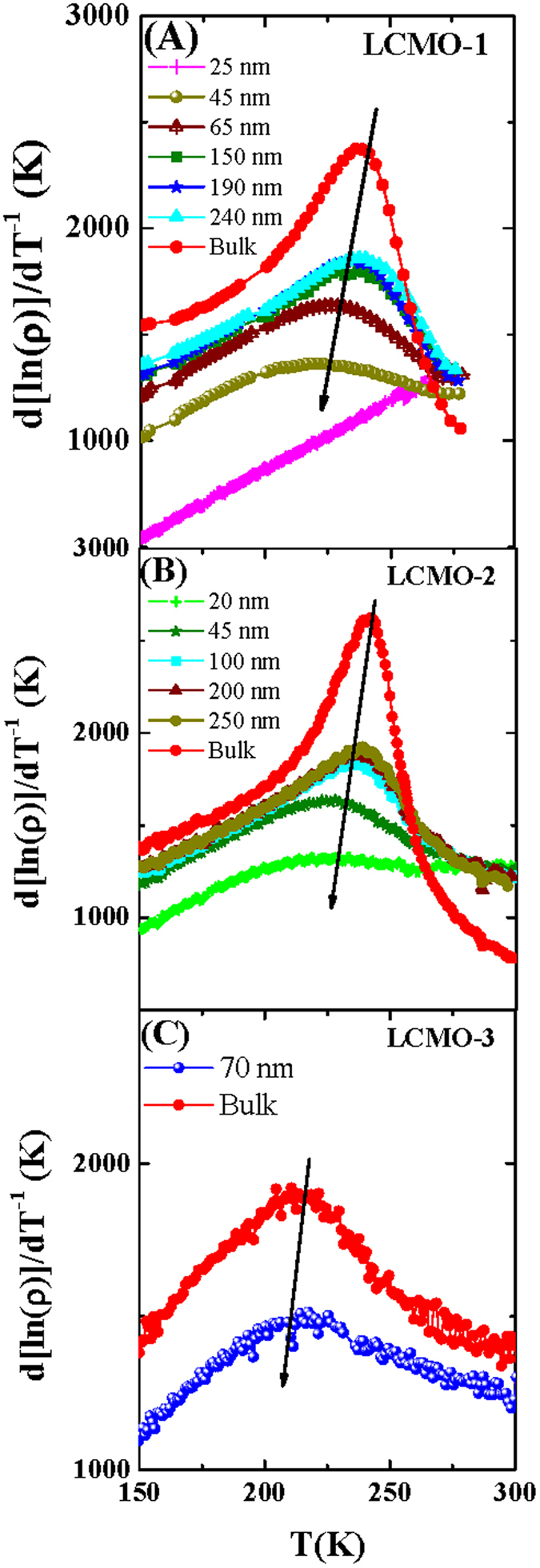
(**A**–**C**) are the temperature variation of d[ln(*ρ*)]/dT^−1^ for LCMO-1, LCMO-2 and LCMO-3 respectively. Peaks of these plot signifying the charge-ordering signature of the compounds which indicated by arrows.

The electrical transport and magneto-transport properties of all nanocrystalline and bulk compounds were measured by the usual four probe method using the home made setup consisting of Oxford magnet system, Keithley voltmeter and current source. Reduced resistivity as a function of temperature in the absence of the external magnetic field for the LCMO-1, LCMO-2 and LCMO-3 is shown in Fig. 4. For each sample the resistivity data was recorded during the cooling from room temperature. With the reduction of temperature resistivity sharply increases below the charge-ordering transition temperature (T_*CO*_). However from the resistivity data the identification of the signature of the charge ordering transition is difficult especially for nanocrystalline compounds. To keep away from this ambiguity the temperature variation of the quantity d[ln(*ρ*)]/dT^−1^ has been plotted for the series LCMO-1, LCMO-2 and LCMO-3 respectively in the Fig. 5(A–C). The peak in the plotted data considered as the charge ordering temperature^16^.

From the Fig. 5 it is clear that the charge-ordering signature is present in all the nanoparticles except the lowest particle size sample of LCMO-1 series (particle size ~25 nm). The d[ln(*ρ*)]/dT^−1^ as a function of temperature plots also indicate that with decreasing the particle sizes, the sharpness of the peak also reduce and ultimately vanish (for ~25 nm sample of LCMO-1 series).

**Figure 6:**
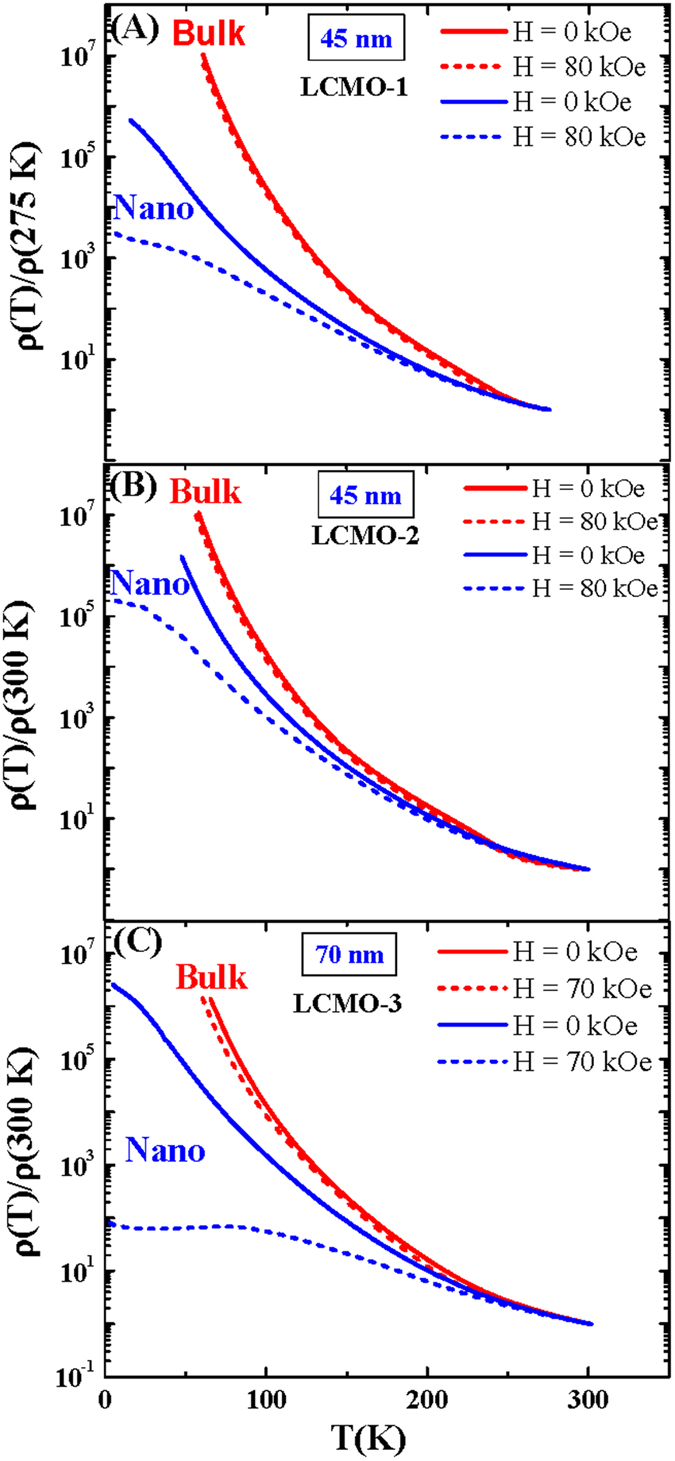
The representative plot of reduced resistivity as a function of temperature of LCMO-1, LCMO-2 and LCMO-3 compounds (bulk and nanoparticles) in the absence and in the presence of external external magnetic field. The plots indicating the drastic decrease of the resistivity in nanoforms of the compounds compared to respective bulk counterparts in the presence of external magnetic field.

The effect of constant external magnetic field on the temperature dependance of electrical resistivity were studied for all the samples. For the sake of clarity, the resistivity as a function of temperature in the absence of and in the presence of a static magnetic field for one representative nanocrystalline compound in each series have been presented in Fig. 6 and the results were compared with their corresponding bulk counterpart.

There are previous report of charge ordered state in nanomaterials of manganite compounds^17^. Charge ordered state can be destroyed with the reduction of the particle sizes^18^,^19^,^20^. In addition to that there are theoretical studies on nanomaterials of manganites that indicate a short ranged ferromagnetic correlation between the uncompensated surface spins is developed with the reduction of the particle size^21^. The resistivity measurement in the presence of external magnetic field (Fig. 6) indicates that the external magnetic field as high as H = 80 kOe was not sufficient to destabilize the robust charge-ordered state of the bulk LCMO-1,−2,−3 compounds. As a result a small change of the resistivity (MR ~20% at T = 100 K, for LCMO-1) due to the application of the 80 kOe magnetic field has been observed. However in contrast to the behavior of the bulk counterpart, the resistivity of the nanocrystalline compounds indicate that it is drastically modified in the presence of the external magnetic field. The weaker charge-ordered state is easily melted and the resistivity is reduced by several orders of magnitude in nanocrystalline compounds in the presence of the external magnetic field at cryogenic temperature.

**Figure 7:**
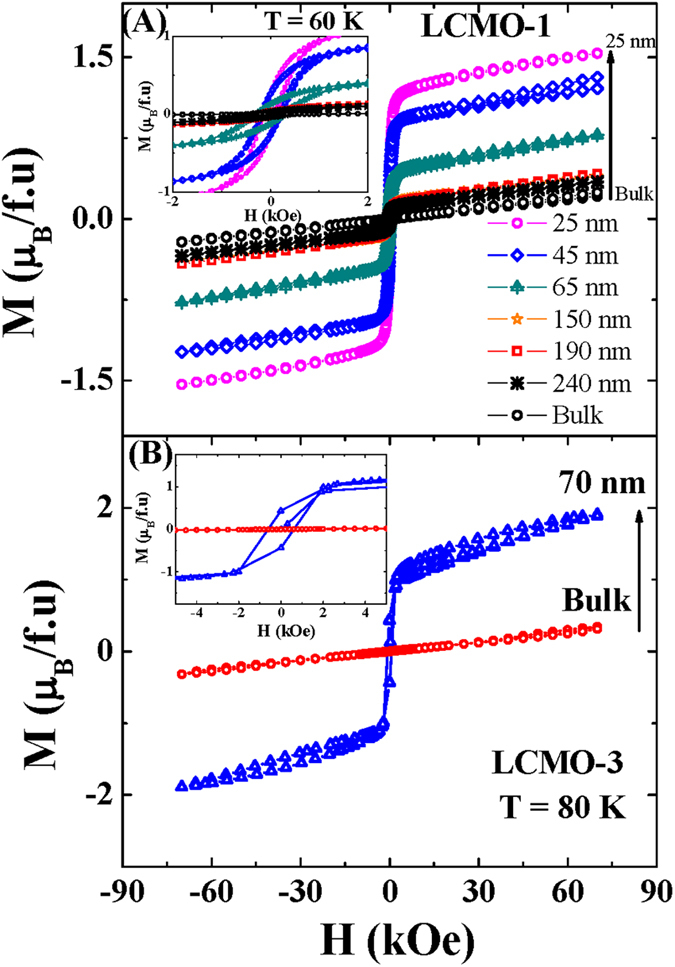
Magnetization as a function of external magnetic field (M(H)) for the series (**A**) LCMO-1 and (**B**) LCMO-3. Insets represent the enlarge portion of the M(H) data at lower magnetic field which indicates the ferromagnetic signature of the compounds in nanoscale regime.

In order to understand the modification of the magnetic properties in case of the nanocrystalline compounds, the magnetization as a function of the external magnetic field at T = 60 K and T = 80 K for the series LCMO-1 and LCMO-3 are respectively presented in Fig. 7. According to the theoretical prediction of Dong *et al*.^22^ for the charge ordered compounds in nanoscale regime, the ferromagnetic component is systematically enhanced with the reduction of the particle size whereas the bulk counterparts indicate the antiferromagnetic nature of the ground state^21^,^22^. In our present case, The ferromagnetic signature in nanoparticles (low field magnetic hysteresis loop) were highlighted in insets of Fig. 7.

The effect of the weakening of the charge-ordering in nanocrystalline compounds was greatly reflected in the magneto-transport measurements. To record the electrical resistance as a function of external magnetic field of all compounds (bulk and nanocrystalline), at first the specified temperature was reached in the absence of any external magnetic field. After reaching the specified temperature electrical resistance of the sample was recorded as a function of external magnetic field. It is worthwhile to be mentioned that the charge ordered antiferromagnetic fraction was easily melted in case of the nanocrystalline compounds (especially below the 100 nm particle size samples) with increasing magnetic field leading to a gigantic decrease of resistance.

**Figure 8:**
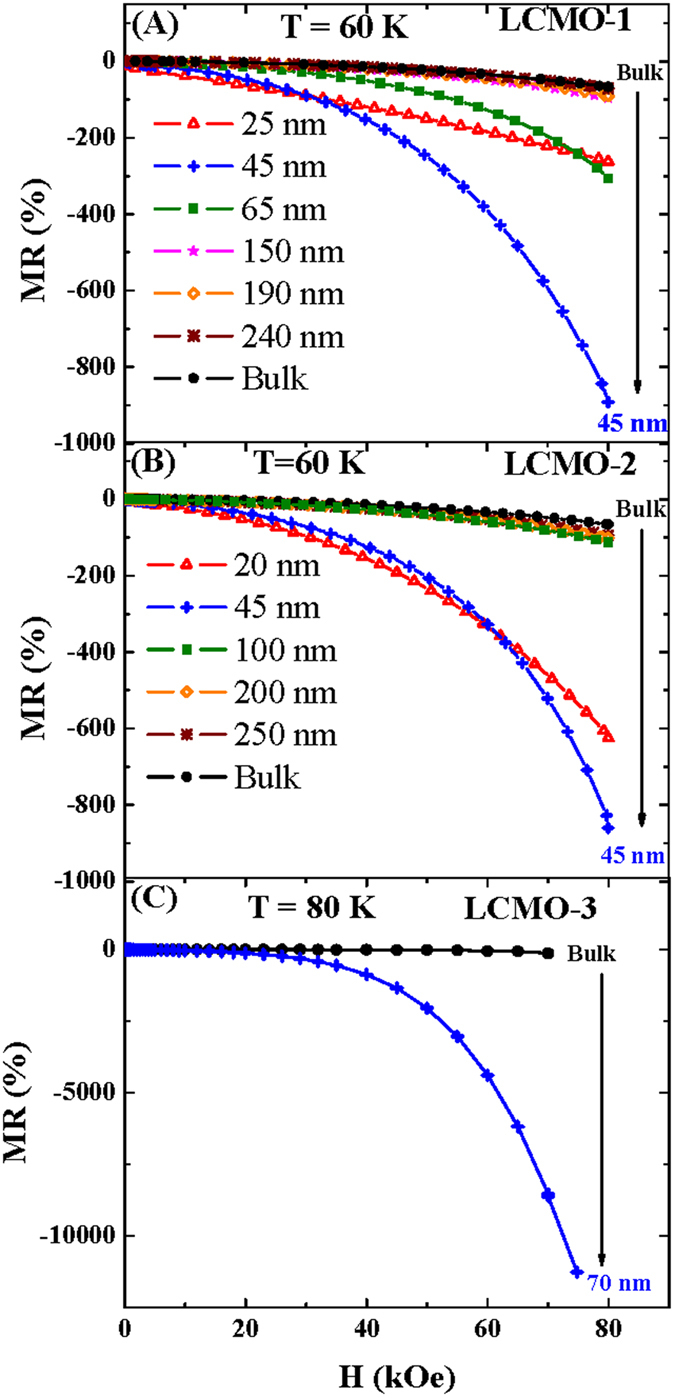
Magnetoresistance as a function of external magnetic field for the series (**A**) LCMO-1 at T = 60 K, (**B**) LCMO-2 at T = 60 K and (**C**) LCMO-3 at T = 80 K. The enhancement of the magnetoresistance with reduction of the particle sizes in each series are indicated by the arrows.

For colossal magnetoresistive materials, it is convenient to define magnetoresistance (MR) as an equation 1.


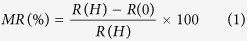


where R(H) is the resistance in the presence of external magnetic field and R(0) is the resistance in the absence of the external magnetic field. The MR of all the samples as a function of external magnetic field at a fixed temperature is shown in Fig. 8. In the context of the behavior of MR it should be mentioned that in each series the MR is greatly enhanced with compared to their bulk counterpart with the reduction of the particle size. From the experimental results of LCMO-1 and LCMO-2 series (shown in Fig. 8) except the lowest particle size, the absolute value of MR as a function of external magnetic field is systematically enhanced with the reduction of the particle size.

**Figure 9:**
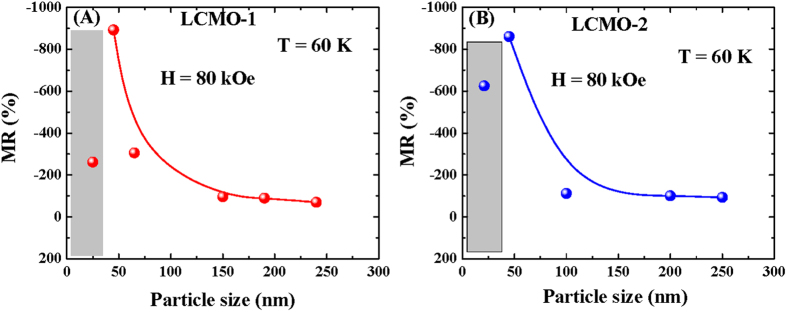
Magnetoresistance as a function of particle size at T = 60 K and H = 80 kOe magnetic field for the series (**A**) LCMO-1 and (**B**) LCMO-2. Lowest size particles shows deviation from the behavior of the rest of the particle sizes, indicated by the shaded area. The solid lines represent guide to the eye.

The particle size dependent MR of the series LCMO-1 and LCMO-2 at H = 80 kOe external magnetic field is shown in Fig. 9. The figure highlights that with the reduction of the particle sizes MR increases systematically. However deviation from the increasing MR behavior was observed for the lowest size (25 nm for LCMO-1 and 20 nm for LCMO-2) particles of both the series. In case of the lowest particle size sample of the LCMO-1 series, the charge-ordering signature was totally absent (Fig. 4). Sharp drop of MR was observed at lower magnetic field and saturates for higher field value similar to a typical ferromagnetic compound (Supplementary Fig. 1 in section-3 of supplementary information part). Whereas in LCMO-2 series, a small charge ordered fraction was present even in lowest particle size sample. This charge-ordered fraction easily melt at lower magnetic field compared to the larger particles in this series. As a result after the melting of the charge-ordering, the rate of suppression of the resistance with the external magnetic field (H > 60 kOe) slows down compared to the 45 nm particle size sample and the MR *vs*. H curve for 20 nm sample cross the curve for 45 nm sample in the LCMO-2 series. The complete flow chart of the magnetoresistances in different magnetic field values for all samples were listed in Table 4.

**Figure 10:**
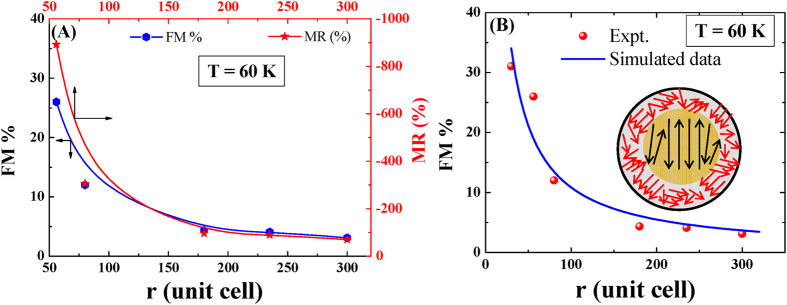
(**A**)Ferromagnetic volume fraction (FM %) and maximum magnetoresistance (MR (%)) as a function of particle size at T = 60 K for the nanocrystalline compounds of the series LCMO-1. The solid lines represent guide to the eye. (**B**) Comparison between the experimentally determined and theoretical simulated ferromagnetic volume fraction (FM %) for the nanocrystalline compounds of LCMO-1 series. Inset of the figure represents the schematic diagram of the phase separated core-shell type structure in nanoscale dimension.

In case of the charge-ordered antiferromagnetic compounds, in nanoscale regime, the spontaneous magnetization which is the signature of ferromagnetism appears due to the pronounced surface effect. With the decreasing particle size, the surface effect increases which leads to the enhanced ferromagnetic volume fraction. In the present study, we observed that the magneto-transport properties of the nanoparticle of the compounds are strongly dependent on the particle size. From our study it emerges that the observed effect is due to the enhancement of surface effect with the reduction of the particle sizes. To generate more clear view the ferromagnetic volume fractions (FM %) and the maximum MR (%) of the nanocrystalline compounds in the series LCMO-1 (excluding lowest particle size) were plotted in Fig. 10(A). The qualitatively similar nature of the FM % and MR (%) as a function of the particle sizes (particle radius in terms of the number of crystalline unit cells, ‘r’) establish the strong correlated nature of the both quantities.

We have determined the FM % from the experimental data of magnetization as a function of external magnetic field [M(H)] at T = 60 K for LCMO-1 series. The magnetization data indicates that at the low magnetic field region, magnetization increases sharply for nanoparticle compounds. The sharp increase of the magnetization is the results of the alignments of the magnetic moments in the shell part in the presence of the small external magnetic field. On the other hand, at the high magnetic field region, the antiferromagnetic responses that is the linear behavior of magnetization as a function of magnetic field of the core part is superimposed on the saturating ferromagnetic part, resulting the non saturating nature of the magnetization up to H = 70 kOe external magnetic field. The fitting of the M(H) isotherms data were performed considering the equation 2.


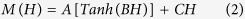


where A, B and C are fitting parameters. By determing the parameters from the experimental M(H) data we have decoupled the antiferromagnetic and ferromagnetic components. To estimate the numerical value of the ferromagnetic volume fraction for different size of the nanoparticles we have used the theoretical saturation value of the magnetization of La_0.48_Ca_0.52_MnO_3_ compound.

The earlier theoretical works reported by Dong *et al*. predicts that for a charge-ordered compound, the phase separated core-shell type structure is energetically stable in nanoscale regime^21^,^22^. In case of the charge ordered compounds, in nanoscale regime, phase separation takes place (a short range ferromagnetic interaction at surface) which is considered as ‘shell’ where as the residual charge ordered (antiferromagnetic) fraction is considered as ‘core’ part. Considering such core-shell type structure in nanoforms of charge ordered manganite compounds, Dong *et al*. derived the analytical relation^22^ (details are given in the Section-2 of supplementary information part).


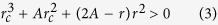


where ‘r_*c*_’ represent the radius of the core part and ‘r’ is the radius of the nanoparticle in terms of the number of unit cells. ‘A’ is the variable parameter which is connected with the nearest neighbor super exchange interaction of Mn-ions (J_*AF*_). The ferromagnetic volume fraction is calculated by the expression


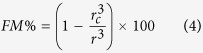


We have analyzed our experimental results keeping the concept of the ‘core-shell’ model in nanoscale regime (the schematic diagram is shown in inset of the Fig. 10(B)). The theoretical simulated results and the experimentally determined FM % of the nanocrystalline compounds (LCMO-1 series) are shown in 10(B) and fairly good agreement was observed (for A = 3.7). From the above concerned theoretical and experimental studies on the charge ordered nanocrystalline compounds indicate the strong correlation between the magnetic and magneto-transport properties. With the systematic increase of FM % in reduced particle sizes, increase of MR (%) was also observed in nanoparticles of LCMO-1, LCMO-2 and LCMO-3 series. If this phenomena is general in nature, magnetic field required to melt the charge-ordered state would also reduce with the reduction of particle size for other charge ordered manganite series. In fact few reports in the literature as mentioned in the Table 5 suggest that the critical magnetic field to melt the charge ordered state of other materials also reduces with lowering the particle size in nanometer scale.

In conclusion, a method for achieving the significant enhancement of magnetoresistance that is the size-induced destabilization of the charge ordered state in nanometer length scale have been discussed in this article. It has been shown (LCMO-3) with the reduction of particle size magnetoresistance at 70 kOe field increased from ~100% to ~10000% at cryogenic temperature. Even at magnetic field as low as 7 kOe, the enhancement of magnetoresistance is from 0.1% to 23%. The significant enhancement of magnetoresistance is the result of the transformation from the robust charge ordered state to the fragile charge ordered state in the nanoparticle. In nanoparticles of charge-ordered manganites, the transformation of charge-ordered insulating state to melted state by the application of much smaller external magnetic field resulted many fold enhancement of magnetoresistance. The experimental results on La_0.48_Ca_0.25_MnO_3_, La_0.46_Ca_0.54_MnO_3_ and La_0.45_Ca_0.55_MnO_3_ compounds illustrate the method of enhancement of MR with the reduction of particle size in nanometer scale. The results were analyzed considering the phase separated core-shell model in nanoscale regime. The weakening of the charge-ordered state and the development of the short range ferromagnetic correlation between the uncompensated surface spins in nanoscale regime is the reason behind this phenomenon. The huge enhancement of magnetoresistance in nanoparticles also has relevance from the technological perspectives as magnetic field sensor. The present phenomenon appears to be wide spread in charge-ordered manganites.

## Methods

The nanocrystalline and bulk La_1−*x*_Ca_*x*_MnO_3_ were prepared by conventional sol-gel method. Starting materials were La_2_O_3_, CaCO_3_, and MnO_2_. Appropriate amounts of pre-heated high pure (99.99%) oxides are converted to their nitrates by using nitric acid and properly dissolved in millipore water. All individual clear water solutions were mixed up and required amount of citric acid was added. The mixture was heated at 80–90 °C by using a water bath until the gel was formed. The gel was decomposed at 200 °C and black porous powder was formed. Same powder was utilized to prepare bulk to nano different size samples by annealing at different temperature and time span. The annealing were performed in air and at atmospheric pressure. For the present study the chemical composition of the all compounds and their short names have been listed in Table 1. For a particular series the chemical composition for all the samples, bulk to nano is identical since the different particle size samples were prepared by the heat treatment at different temperature and time duration of the same sol-gel prepared powder sample.

## Additional Information

**How to cite this article**: Das, K. *et al*. Significant enhancement of magnetoresistance with the reduction of particle size in nanometer scale. *Sci. Rep*. **6**, 20351; doi: 10.1038/srep20351 (2016).

## Supplementary Material

Supplementary Information

## Figures and Tables

**Table 1:** Composition and short name of the compounds.

Compound	Short name
La_0.48_Ca_0.52_MnO_3_	LCMO-1
La_0.46_Ca_0.54_MnO_3_	LCMO-2
La_0.45_Ca_0.55_MnO_3_	LCMO-3

**Table 2:** Particle sizes of LCMO-1.

Annealing temperature (°C)	Annealing time (hr.)	Average particle size (nm)
600	4	25
800	4	45
900	3	65
1000	3	150
1000	6	190
1000	10	240
1300	36	Bulk (>1 *μ*m)

**Table 3:** Particle sizes of LCMO-2.

Annealing temperature (°C)	Annealing time (hr.)	Average particle size (nm)
800	4	20
900	3	45
1000	3	100
1000	6	200
1000	10	250
1300	36	Bulk (>1 *μ*m)

**Table 4:** Particle size dependent magnetoresistance in the presence of different external magnetic field.

Compound	Particle size	MR (%)				
		H = 5 kOe	H = 10 kOe	H = 20 kOe	H = 50 kOe	H = 70 kOe
LCMO-1	25 nm	−27	−38	−66	−150	−221
	45 nm	−11	−21	−47	−244	−574
	65 nm	−2.5	−5.3	−14.6	−77	−196
	150 nm	−0.43	−2.05	−6.42	−34	−71
	190 nm	−0.34	−1.65	−5.63	−30	−62
	240 nm	−0.3	−1.52	−4.64	−25	−51
	Bulk (>1 *μ*m)	−0.24	−0.69	−3.41	−22	−48
LCMO-2	20 nm	−14.2	−26	−51	−239	−470
	45 nm	−7	−16.5	−39	−206	−521
	100 nm	−1.2	−2.64	−9	−40	−80.5
	200 nm	−1.17	−2.49	−8.39	−38.3	−73
	250 nm	−1.13	−1.68	−6.94	−37	−68
	Bulk (>1 *μ*m)	−0.05	−0.43	−3.19	−23	−48
LCMO-3	70 nm	−15	−37	−128	−2047	−8536
	Bulk (>1 *μ*m)	−0.02	−0.6	−2.84	−20	−131

**Table 5:** Reduction of critical magnetic field in nanoparticles.

Compound	Critical field (H_*C*_)		Ref.
	Bulk	Nano (~40 nm)	
Pr_0.5_Ca_0.5_MnO_3_	27 Tesla	5.5 Tesla	13,14
Nd_0.5_Ca_0.5_MnO_3_	20 Tesla	6 Tesla	2,14,23
