# Supplementary material for: Significant enhancement of magnetoresistance with the reduction of particle size in nanometer scale
Source: Sci Rep. 2016 Feb 3;6:20351. doi: 10.1038/srep20351 (PMC4738252; doi:10.1038/srep20351)
Supplement: Supplementary Information [file srep20351-s1.pdf]

Significant enhancement of magnetoresistance with the reduction of particle size in nanometer scale

Kalipada Das, P. Dasgupta, A Poddar, and I. Das*
CMP Division, Saha Institute of Nuclear Physics,
1/AF, Bidhannagar, Kolkata 700 064, India

January 8, 2016

1 Supplementary information

1.1 Section-1

According to the Scherrer's formula, the average particle size (d) of nanocystalline compound is given by

$$d = \frac{K\lambda}{\beta \cos\theta} \tag{1}$$

Here $K \sim 0.9$ (constant) and $\lambda = 1.54$ Å(wave length of x-ray). Effective full width at half maxima (FWHM), β is calculated using the relation

$$\beta = B - \frac{b^2}{B} \tag{2}$$

Where 'B' and 'b' are FWHM of a peak of the nanocrystalline sample at a particular angle of diffraction and FWHM of the corresponding peak of the bulk form of that sample (measured in the same instrument)

1.2 Section-2

According to the theoretical report on the phase separated (PS) charge-ordered nanoparticle (*Appl. Phys. Lett.,* **2007**, 90, 082508), when a charge-ordered core is wrapped by a FM shell, the energy (E_{PSnano}) can be expressed by the equation 3.

$$E_{PSnano} = \frac{4}{3}\pi r_c^3 E_{CO} + 4\pi r_c^2 \frac{J_{AF}}{6} + \frac{4}{3}\pi(r^3 - r_c^3)E_{FM} - 4\pi(r^2 + r_c^2)\frac{J_{AF}}{2} \quad (3)$$

Where E_{CO} and E_{FM} represent the energy per unit volume of the CO and the FM part respectively. The total and core radii of the nanoparticle are represented by r and r_c respectively. The parameter, J_{AF} is the super exchange interaction.

However the energy (E_{COnano}) of a spherical nanoparticle of pure chrage-ordered state can be represented by equation 4

$$E_{COnano} = \frac{4}{3}\pi(r^3 E_{CO} + \frac{1}{2}r^2 J_{AF}) \quad (4)$$

The energy difference between the pure charge-ordered and phase-separated state is

$$\delta E = E_{COnano} - E_{PSnano} \quad (5)$$

$$\delta E = \frac{4}{3}\pi[(r^3 - r_c^3)(E_{CO} - E_{FM}) + (2r^2 + r_c^2)J_{AF}] \quad (6)$$

For a stable phase-separated state in nanoscale regime $\delta E > 0$. From the equation 6, for a stable FM shell requires

$$r_c^3 + Ar_c^2 + (2A - r)r^2 > 0 \quad (7)$$

Where $A = J_{AF}/(E_{FM}$-$E_{CO})$ is the variable parameter which is connected with the nearest neighbor super exchange interaction of Mn-ions (J_{AF}). The ferromagnetic volume fraction is calculated by the expression

$$FM\% = (1 - \frac{r_c^3}{r^3}) \times 100 \quad (8)$$

1.3 Section-3

In our present study it was observed that the enhancement of the magnetoresistance is appears even at low magnetic field region with the reduction of the particle sizes in nanometer scale. The enlarged view of the external magnetic field dependent magnetoresistance in the lower magnetic field range for all the nanoparticles are given in Figure-1.

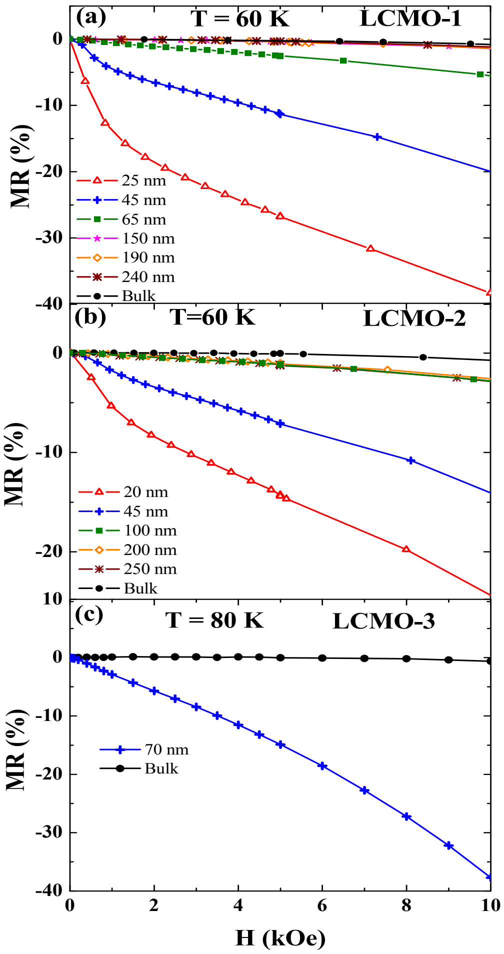

Figure 1: Enlarged view of the magnetoresistance as a function of external magnetic field at low magnetic field region for the series (a) LCMO-1 at T = 60 K, (b) LCMO-2 at T = 60 K and (c) LCMO-3 at T = 80 K.

1.4 Section-4

To determined the average particle sizes, the Transmission Electron Microscopy (TEM)study have been carried out. In addition to that, we have also done the Scanning Electron Microscopy (SEM) study for the nanocrystalline compounds. As a demonstrative representation the average particle size determination of the nanoparticle having average particle size 70 nm (LCMO-3 series) from the different studies is given below.

The TEM picture of the nanocrystalline compound (LCMO-3 series) along with the histogram of the particle size distribution is given in the Figure-2. From the particle size distribution histogram the average particle size found to 70 nm.

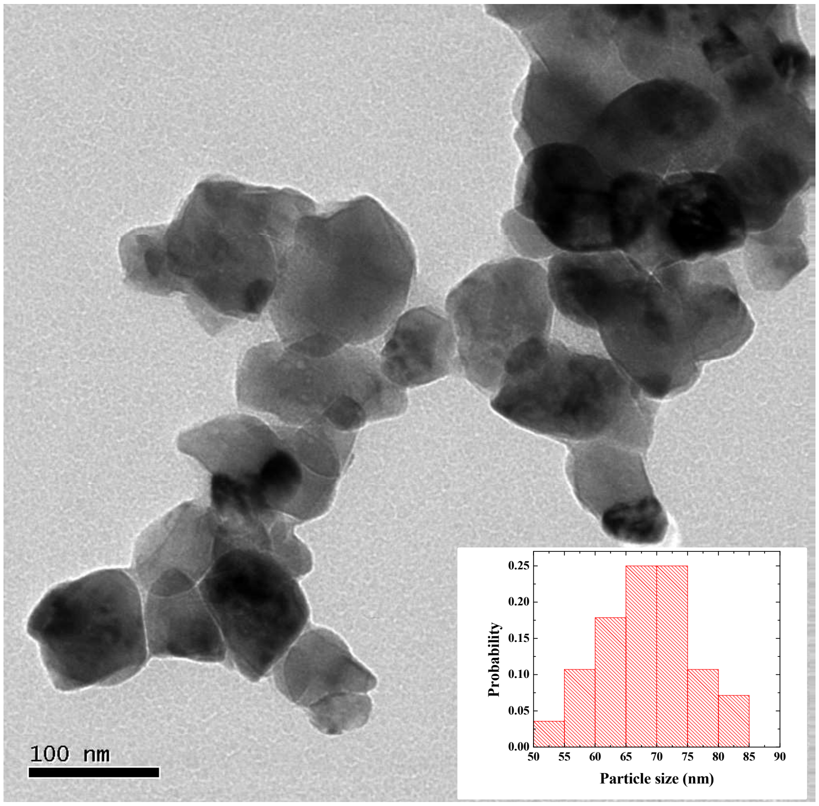

Figure 2: Transmission Electron Microscopy (TEM) image of the nanocrystalline compound having average particle size 70 nm. Inset indicates the histogram of the particle size distribution.

Similar as TEM study, we have estimated the average particle size from the Scanning Electron Microscopy (SEM)study. The SEM image along with the particle size distribution curve of the same nanocrystalline compound is given in Figure-3 (the average particle size ∼65 nm).

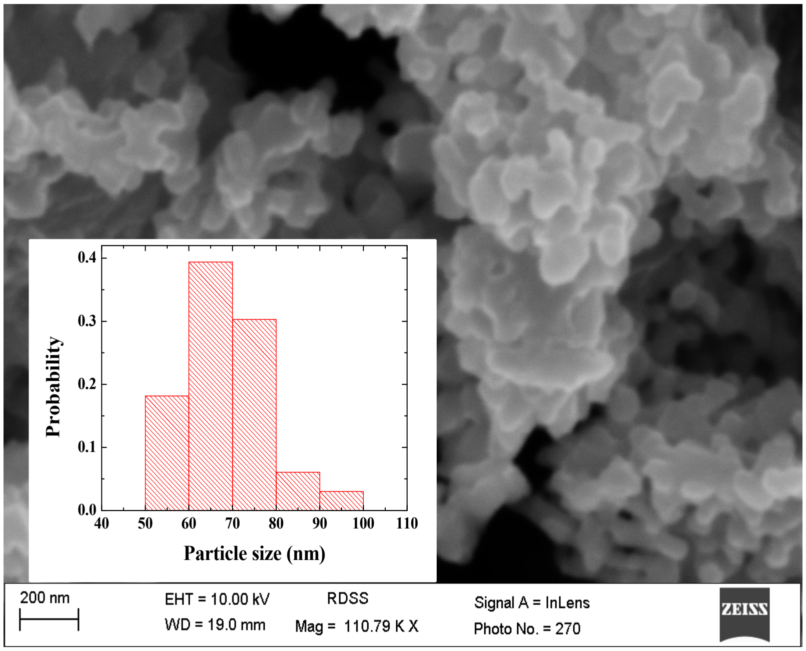

Figure 3: Scanning Electron Microscopy (SEM) image of the nanocrystalline compound having average particle size 70 nm as determined from TEM. Inset indicates the histogram of the particle size distribution.

In addition to that, as mentioned in the Section-1 of the supplementary information part, the calculated average particle size from the x-ray diffraction data of the LCMO-3 series is found to ∼ 60 nm.

The crystallinity in nanoparticles may also influence the magneto-transport properties. In our present case, the High Resolution Transmission Electron Microscopy (HRTEM) image of nanoparticle (average particle size 70 nm) indicate the well crystalline nature. One representative HRTEM image is given in Figure-4.

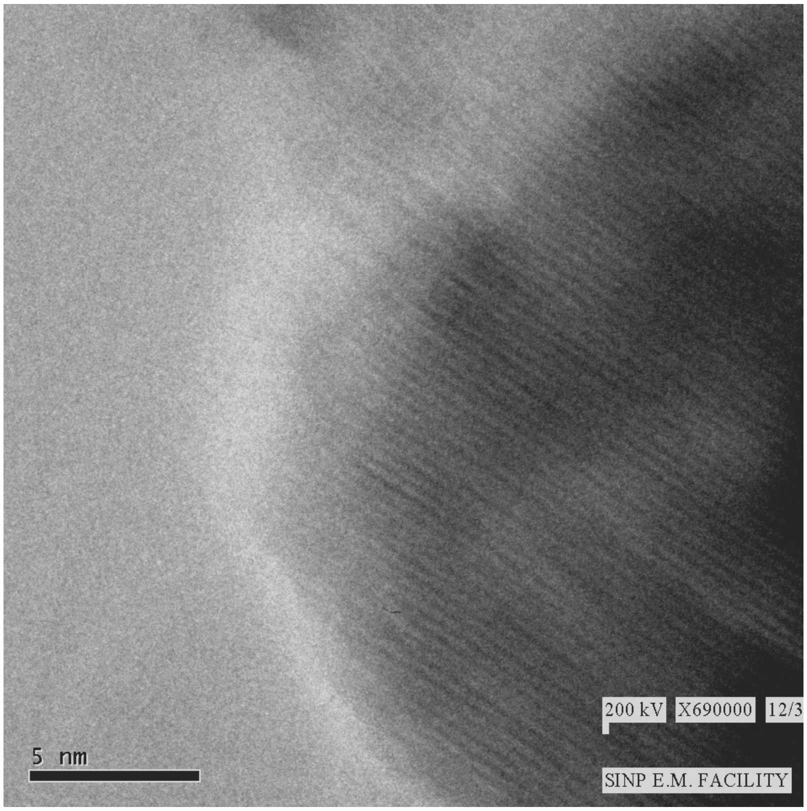

Figure 4: High Resolution Transmission Electron Microscopy (HRTEM) image of the nanocrystalline compound having average particle size 70 nm.

1.5 Section-5

It is emphasized in different earlier studies that oxygen non-stoicheometry can influence the physical properties of the manganites. The electron conduction and magnetic interaction can be significantly modified due to the presence of oxygen vacancies. Such changes are usually manifested in transitions. In some cases, one characteristic transition can even be hindered due to this oxygen non-stoicheometry (*J. Phys.: Condens. Matter*, **24**, 366004(2012)). In the present study, there is no significant change in charge ordering transition temperatures of nanoparticles in comparison with bulk. We have also performed the x-ray photoelectron spectroscopy (XPS) measurements on bulk and nanocrystalline compounds. As a representative example, we

have included the XPS data of $La_{0.48}Ca_{0.52}MnO_3$ compound (bulk and one nanoparticle ($\sim$ 65 nm))in Figure-5.

According to the chemical composition of the $La_{0.48}Ca_{0.52}MnO_3$ compound, the ratio of the Mn^{4+} and Mn^{3+} ion is 1.0833 (52/48 = 1.0833). Our experimental results of XPS measurements indicate that the ratio of Mn^{4+} and Mn^{3+} for bulk is 1.084 and for nanoparticle, it is 1.089. The experimental observed values are very close to above mentioned value (1.0833) for the $La_{0.48}Ca_{0.52}MnO_3$ compound. In this context it can be mentioned that the bulk and the nanoparticles were prepared using the same powder of LCMO, just by heating at different temperature and time duration as discussed in the manuscript. Hence it may assume that the variation of the particle size played the vital role behind the phenomena addressed in the manuscript.

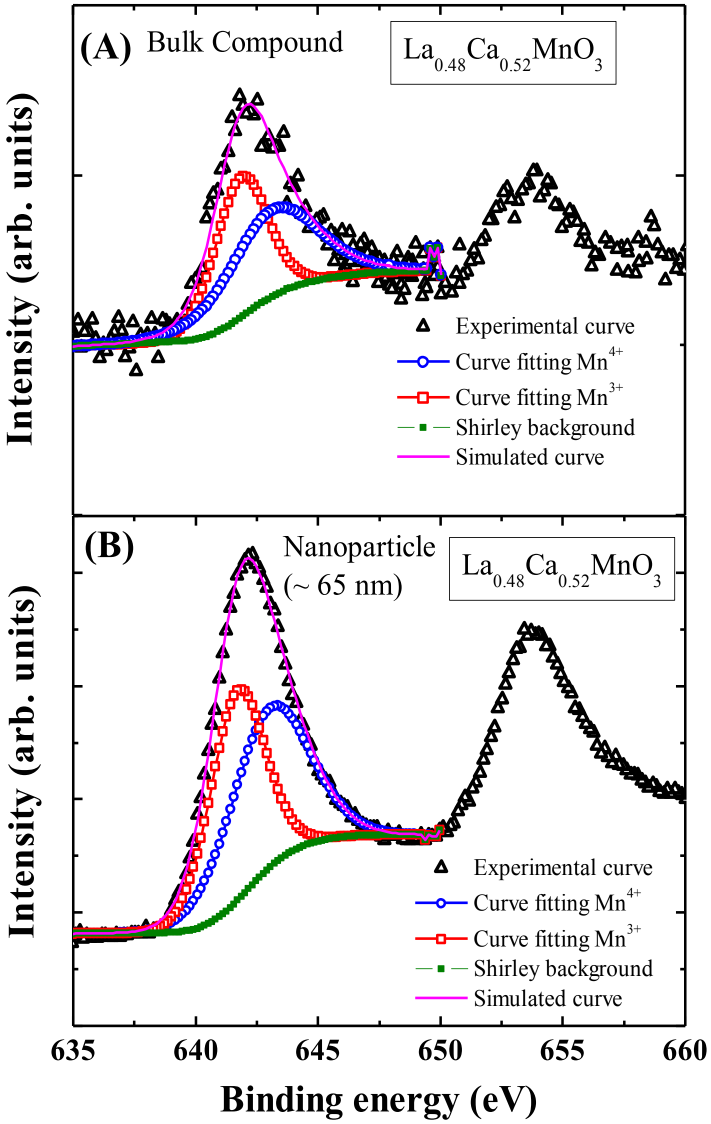

Figure 5: XPS spectrum with fitted curves (Mn $2P_{3/2}$ peak) for (A) bulk compound and (B) nanoparticles having average particle size 65 nm.
